# The use of fish and herptiles in traditional folk therapies in three districts of Chenab riverine area in Punjab, Pakistan

**DOI:** 10.1186/s13002-020-00379-z

**Published:** 2020-06-24

**Authors:** Muhammad Altaf, Arshad Mehmood Abbasi, Muhammad Umair, Muhammad Shoaib Amjad, Kinza Irshad, Abdul Majid Khan

**Affiliations:** 1 Department of Zoology, Women University of Azad Jammu and Kashmir, Bagh, Pakistan; 2 Department of Environment Sciences, COMSATS University Islamabad, Abbottabad Campus, Abbottabad, 22060 Pakistan; 3 School of Agriculture and Biology, Shanghai Jiao Tong University, Shanghai, China; 4 Department of Botany, Women University of Azad Jammu and Kashmir, Bagh, Pakistan; 5 Department of Zoology, University of the Punjab, Lahore, Pakistan

**Keywords:** Ethnozoology, Medicinal, Herptile, Fish, Punjab, Pakistan

## Abstract

**Background:**

Like botanical taxa, various species of animals are also used in traditional and modern health care systems. Present study was intended with the aim to document the traditional uses of herptile and fish species among the local communities in the vicinity of the River Chenab, Punjab Pakistan.

**Method:**

Data collected by semi-structured interviews and questionnaires were subsequently analyzed using relative frequency of citation (FC), fidelity level (FL), relative popularity level (RPL), similarity index (SI), and rank order priority (ROP) indices.

**Results:**

Out of total 81 reported species, ethnomedicinal uses of eight herptiles viz. *Aspideretes gangeticus*, *A*. *hurum*, *Eublepharis macularius*, *Varanus bengalensis*, *Python molurus*, *Eryx johnii*, *Ptyas mucosus mucosus*, *Daboia russelii russelii* and five fish species including *Hypophthalmichthys molitrix*, *Cirrhinus reba*, *Labeo dero*, *Mastacembelus armatus*, and *Pethia ticto* were reported for the first time from this region. Fat, flesh, brain, and skin were among the commonly utilized body parts to treat allergy, cardiovascular, nervous and respiratory disorders, sexual impotency, skin infections, and as antidote and anti-diabetic agents. *Hoplobatrachus tigerinus*, *Duttaphrynus stomaticus*, and *Ptyas mucosus mucosus* (herptiles), as well as *Labeo rohita*, *Wallago attu*, and *Cirrhinus reba* (fish) were top ranked with maximum informant reports, frequency of citations, and rank order priority. *Uromastyx hardwickii*, *Ctenopharyngodon idella*, *H*. *molitrix*, *Cirrhinus mrigala*, *C*. *reba*, *L*. *rohita*, *L*. *calbasu*, *L*. *dero*, and *Pethia ticto* were the species with 100% fidelity level. Furthermore, medicinal uses of *Aspideretes gangeticus*, *Aspideretes hurum*, *Calotes versicolor*, *Daboia russelii russelii*, *Hypophthalmichthys molitrix*, *Cirrhinus reba*, *Labeo dero*, *Mastacembelus armatus*, *Pethia ticto*, and *Gagata cenia* were reported for the first time.

**Conclusion:**

About half of the reported species depicted zero similarity index with previously reported literature, which indicates strong associations of local inhabitants with animal species, particularly for therapeutic purpose. Inclusive studies on composition and bioactivities of the species with maximum use reports may contribute significantly in animal-based novel drugs discovery.

## Introduction

The multipurpose usage of animal species, e.g., as food, medicine, entertainment, magic, music and religion, tools for art, and in trade, is well known [1–9]. It has been reported that loving, watching, and working with animal species is beneficial to lower heartbeat and control stroke [10]. Animal-based products are used as traditional medicines, and an estimated 8.7% of the vital chemicals used in modern healthcare systems are extracted from different animal species [11]. However, compared to plant species, animal-based products are widely neglected [12]. Inhabitants of rural areas are more depended on animal-based products as food and medicines, and possess significant traditional knowledge of zootherapies [9, 11, 13]. Many species of animals, either wild or domesticated, are important to humans [13]. Wild animal species are often under threat due to anthropogenic activities like illegal hunting and trade for food, medicines, and ornamental purposes, deforestation, agriculture intensification, urbanization, and industrialization [3, 14–19].

Herptiles and fish are recognized as extremely fascinating and important animal species [20–22]. In many societies, different species of herptiles and fish are used in ethnomedicine and folklore to treat health disorders [16, 22, 23]. An estimated 10,450 species of reptiles and 7850 amphibian species have been reported [24] globally. In Pakistan, 195 species of reptiles [25] and 24 species of amphibians [26] have been documented so far. The Asian region has also a high diversity of marine and freshwater fish species (22907 and 10036 species, respectively) [24, 27]. An estimated 186 species of freshwater fish and 719 species of marine water fish have been reported so far in Pakistan [28]. However, traditional uses of animal species, particularly to treat diseases in humans and other animals, have rarely been documented in Pakistan [6, 7, 29–32]. To best of our knowledge, traditional uses of herptiles and fish species have never been reported before in Pakistan. Therefore, the present study was planned to document herptiles and fish species used to treat various diseases by the local communities residing along the Chenab riverine areas, i.e., Gujranwala, Gujrat, and Sialkot districts in the Punjab province of Pakistan. Qualitative indices were used to elucidate commonly utilized species with high fidelity level and frequency of citation. We hope the data provided will be of significant value for pharmaceutical industries to discover animal-based novel drugs to meet the recent challenges to human health.

## Materials and methods

### Study area

**Fig. 1:**
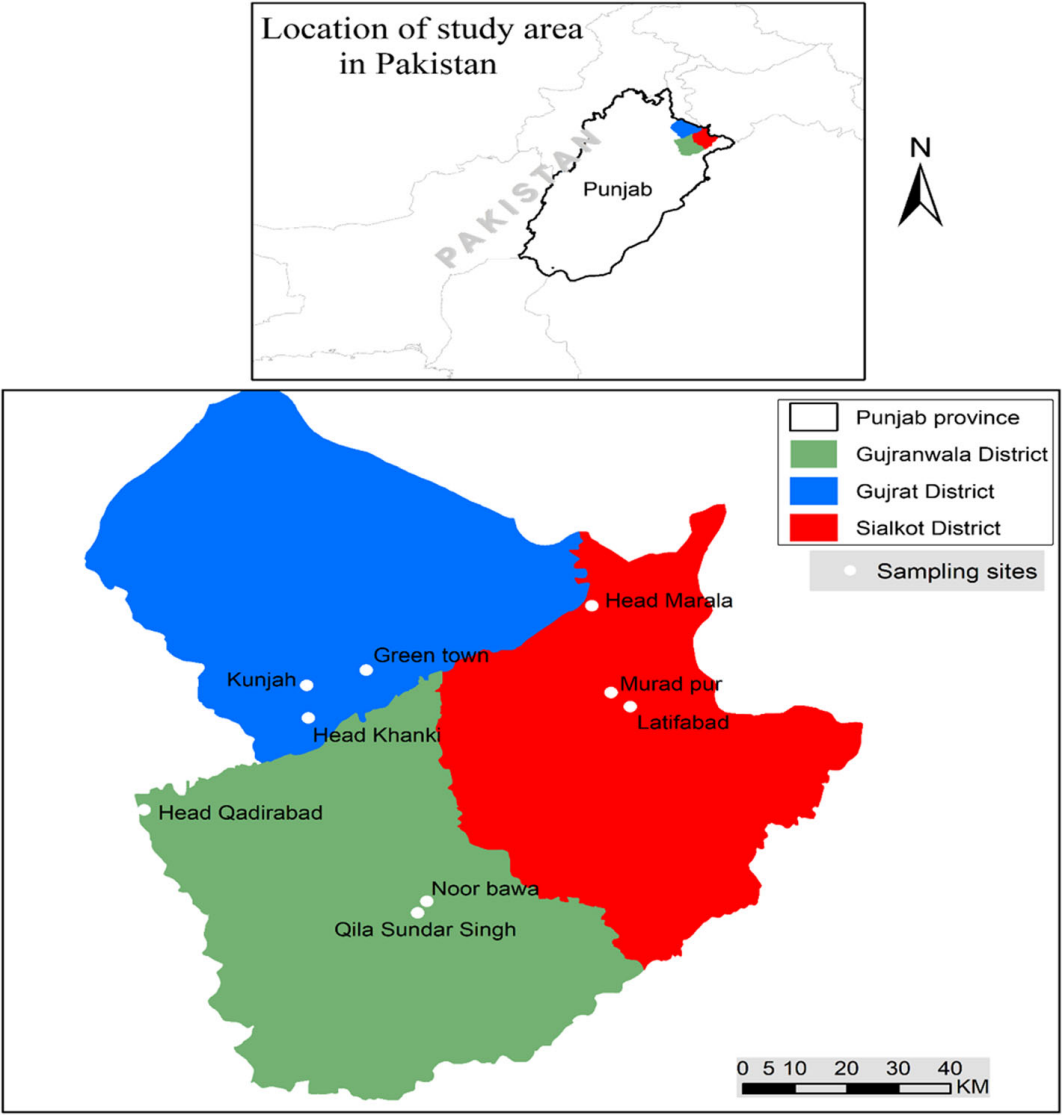
Map of study area with survey sites

The River Chenab is the combination of two main streams, i.e., Chandra and Bhaga originating from the Himalayan region of Himachal Pradesh in India. After passing through the Siwalik Range in the south, and the Lesser Himalayas in the north of Indian Jammu and Kashmir, it continues into Pakistan [33]. The present study was conducted in three districts of the Chenab riverine area, i.e., Gujranwala, Gujrat, and Sialkot (Fig. 1) from March 2016 to April 2017. The study area covers 9830 km^2^, with temperature ranges from around 0 °C in December to 50 °C in June [34–36]. This region has a high diversity of wild fauna, comprising 150 species of birds, 47 herptiles, 34 fish, and 15 mammalian species [15, 37–39]. Demographically almost 52% of the population are male, and 48% are female. The major population is rural, and encompasses Arain, Gujjar, Jutt, Sheikh, Rana Butt, Malik, and Mughal casts. Punjabi is the common language spoken, although some people speak Siraiki and Urdu, while educated people can also speak English to some extent [34–36].

### Data collection and analysis

Following the Nagoya Protocol, prior informed consent was taken from local informants for data collection and publication. In addition, the International Society of Ethnobiology Code of Ethics (http://www.ethnobiology.net/) was also followed. Ethnomedicinal uses of herptiles and fish species along with cultural importance were collected from local informants (*n* = 100) using semi-structured interviews and group discussions. Informants including farmers, fishermen, hunters, teachers, and health practitioners were selected based on their traditional knowledge of animal species, i.e., herptiles and fish. Animals were identified using “The Amphibian and Reptiles of Pakistan” [25], and “Freshwater Fish of Pakistan” were also consulted for correct classification and identification of fish of the study area [40]. Different indices, i.e., relative frequency of citation (RFC), fidelity level (FL), relative popularity level (RPL), rank order priority (ROP) and similarity index (SI), were used to analyze that data.

Relative frequency of mention (RFC) was calculated using formula as reported previously [41].

$$ \mathrm{RFC}=\frac{\mathrm{FC}}{\mathrm{N}}\ \left(0\le \mathrm{RFC}\le 1\right) $$

Where FC is the frequency of citation for an ethnomedicinal or cultural use of a specific species and *N* is the total number of informants.

Fidelity level (FL) was obtained using the method explained earlier [42] based on formula

$$ \mathsf{FL}\ \left(\%\right)={\mathsf{N}}_{\mathsf{p}}/\mathsf{FC}\times \mathsf{100} $$

where *N*_p_ indicates number of informants reporting major ailment for a specific species of herptiles or fish and FC is the frequency of citation for ethno-medicinal or cultural use of that species.

Relative popularity level (RPL) of the reported species was elucidated as reported by [43, 44]. Herptiles and fish species were classified into two groups (i) “popular” and (ii) “unpopular.” Popular herptiles and fish species were those having more than half of the maximum frequency of citation (FC), whereas the left-over herptiles and fish were documented as unpopular. For popular herptiles and fish species, a horizontal line was imaginary, namely the average number of uses per species is independent of the frequency of citation (FC), who recognizes the herptiles and fish; therefore, the average numeral of uses of a popular herptile and fish species does not enhance with the add to frequency of citations who cite a herptile and fish for any medical use. For the popular herptiles and fish, the RPL was chosen to one (1). For herptiles and fish in the unpopular group, the relative popularity level value is less than 1.0.

Rank order priority (ROP) is used to grade plants and animal species and was calculated as explained earlier [43, 44] and was analyzed by the following formula

$$ \mathrm{ROPs}=\mathrm{FL}\times \mathrm{RPL} $$

Similarity index (SI) was calculated as reported previously [3]

$$ \mathsf{SI}={\mathit{\mathsf{M}}}_{\mathsf{s}}/{\mathit{\mathsf{M}}}_{\mathit{\mathsf{t}}}\kern0.75em \left(0\le \mathrm{SI}\le 1\right) $$

*M*_s_ = Alike number of medicinal uses in the previous and present research records for a specific herptiles and fish species. *M*_t_ = Total number of medicinal uses in the present research reports for a specific herptiles and fish species.

Principal component analysis (PCA). Data were statistically analyzed with the help of principal component analysis by using Past software Version 3 [45].

## Results and discussion

### Demography

**Fig. 2:**
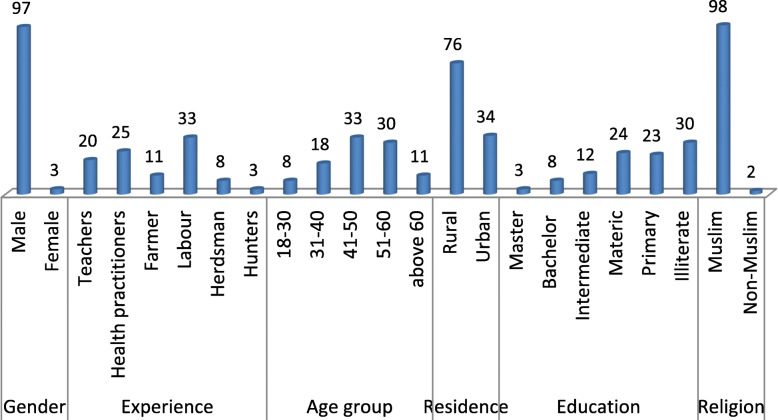
Ethnographic data of local informants

Data were collected from 100 informants of an age between 18 and 75 years (Fig. 2). About 70% informants were literate, and participants had finished having primary, matric, intermediate, bachelors, and master levels (23, 24, 21, 8, and 3, respectively). The majority of the informants (76%) were from rural areas with agricultural background.

### Local nomenclature

Vernacular names of animal species are usually based on environment, myths, morphological characteristics, habitat, and social associations of species with humans. As mentioned in Table 1, “daddo” is used as suffix in six animals (13%) of the reported herpetiles such as *Bufotes latastii* (chitkbra daddo), *Duttaphrynus stomaticus* (ghariallo daddo), *Microhyla ornate* (bona daddo), *Fejervarya limnocharis* (pidda daddo), *Hoplobatrachus tigerinus* (wada daddo), and *Sphaerotheca breviceps* (chota dahri daddo). Variations in the vernacular names of these animals are due morphological differences, e.g., *H*. *tigerinus* has a larger size and was called “wada daddo.” Similarly, *M*. *ornata* has a smaller size and was called “bona daddo.” A very small frog was called “pidda daddo,” while *B*. *latastii*, which has patches on body, was called “chitkbra daddo,” and *S*. *breviceps*, *Amphiesma stolatum*, and *Ophisops jerdonii*, which all have lines on the body, were named as “chota dahri daddo.”

**Table 1:** Comparative assessment of present and previously reported ethnomedicinal uses of herptiles and fishes

S#	Scientific, common and local name	Parts used	MA	Diseases	Code	IMA	ND	FC	FL	RPL	ROP	Reported use	References	SI
**Herptiles**														
1	*Duttaphrynus stomaticus* (Lütken,1864), Indus Valley toad, Ghariallo daddo	Skin	T	Skin infection in animals	BF1	3	1	30	10.0	0.667	6.67	Allergy, pneumonia, dermatitis, ripened abscess, wounds	[46–48]	0
2	*Hoplobatrachus tigerinus* (Daudin, 1802), Indian Bullfrog, Wada daddo	Fat, oil	T	**Sexual potency**	HT1	2	3	40	5.0	0.889	4.44	Acidity, burn, cold, cough, diarrhea, dysentery, wound	[49–52]	0
				**Backbone pain**	HT2	2			5.0		4.44			
				**Joint pain**	HT3	2			5.0		4.44			
3	*Aspideretes gangeticus* Ernst and Barbour, 1989, Indian soft shell, Plaither	Flesh ash, fat, oil	O, T	**Skin diseases**	AG1	2	3	8	25.0	0.178	4.44			0
				**Piles**	AG2	2			25.0		4.44			
				**Sexual potency**	AG3	4			50.0		8.89			
4	*Aspideretes hurum* Ernst and Barbour, 1989, Peacock soft shell, Kachhokuma	Fat, oil	T	**Sexual potency**	AH1	4	3	8	50.0	0.178	8.89			0
				**Backbone pain**	AH2	2			25.0		4.44			
				**Joint Pain**	AH3	2			25.0		4.44			
5	*Lissemys punctata andersoni*Webb, 1980, Indian Flap-shelled Turtle, Hara kachopra	Carapace Ash, fat, oil	O, T	**Internal injuries**	LP1	1	4	7	14.3	0.156	2.22	Allergy, acne, piles, arthritis, asthma, bronchitis, burns, cough, dermatitis, epilepsy, backbone pain,diabetes, urinary obstruction, diarrhea, indigestion, lung diseases, malaria fever, menorrhagia, rashes, sexual dysfunction, wounds, tuberculosis	[48, 53–55]	0.75
				**Allergy**	LP2	1			14.3		2.22			
				**Cough**	LP3	2			28.6		4.45			
				**Sexual potency**	LP4	4			57.2		8.90			
6	*Calotes versicolor* (Daudin, 1802), Oriental garden lizard, Girgit	Flesh ash	T	**Foot and toes injuries**	CV1	2	1	12	16.7	0.267	4.45			0
7	*Laudakia agrorensis* (Stoliczka, 1872), Agror agama lizard, Goh	Fat, oil, bile	T	Joint pain	LA1	2	4	20	10.0	0.444	4.44	Arthritis, burn, cough, fever, jaundice, malaria, sexual stimulant, skin disease	[47, 48, 50, 52, 56–58]	0.5
				Sexual potency	LA2	3			15.0		6.67			
				Snake, spider, wasp and scorpion sting	LA3	4			20.0		8.89			
				Body pain	LA4	2			10.0		4.44			
8	*Eublepharis macularius* (Blyth, 1854), Common leopard gecko, Korh kirli	Fat	T	Cancer	EM1	1	1	6	16.7	0.133	2.23			0
9	*Uromastyx hardwickii* Smith, 1935, Indus spiny-tailed lizard, Sanda	Fat, oil	T	Joint pain	UH1	20	4	24	83.3	0.533	44.44	Enhance sexual power, treat earache, backbone pain, joint pain, headache	[48, 59]	0.5
				Body pain	UH2	20			83.3		44.44			
				Sciatica pain	UH3	4			16.7		8.89			
				Sexual potency	UH4	24			100		53.33			
10	*Varanus bengalensis* (Daudin, 1802), Bengal Monitor, Bengali goh, Goh	Fat, oil	T	**Joint pain**	VB1	3	4	20	15.0	0.444	6.67			0
				**Sexual potency**	VB2	2			10.0		4.44			
				**Snake, spider, wasp and scorpion sting**	VB3	4			20.0		8.89			
				**Body pain**	VB4	3			15.0		6.67			
11	*Python molurus* (Linnaeus, 1758), Rock pathon, Azdha sap	Fat, oil	T	**Wound**	PM1	3	3	5	60.0	0.111	6.67			0
				**Joint pain**	PM2	3			60.0		6.67			
				**Sexual potency**	PM3	3			60.0		6.67			
12	*Eryx johnii* (Russell, 1801), Indian Sand boa, Do moi	Oil	T	**Leucoderma**	EJ1	2	2	22	9.1	0.489	4.44			0
				**Sexual potency**	EJ2	2			9.1		4.45			
13	*Ptyas mucosus mucosus* (Linnaeus, 1758), Indian rat snake, Chohay-maar sap	Skin	T	**Eyesight**	PMM1	3	1	25	12.0	0.556	6.67			0
14	*Naja naja naja* (Linnaeus, 1768), Indian cobra, Kala naag	Fat, skin, oil	T	Sciatica	NNN1	2	4	17	11.8	0.378	4.44	Arthritis, cancer, eye sight, leprosy, muscular pain, sexual weakness, sciatica, snakebite	[48, 51, 52, 59]	1
				Snakebite	NNN2	4			23.6		8.92			
				Eye sight	NNN3	7			41.2		15.56			
				Sexual weakness	NNN4	2			11.8		4.46			
15	*Daboia russelii russelii* (Shaw and Nodder, 1797), Russell's chain viper, Dabian wala sap	Fat, oil	T	**Urine problem**	DRR1	1	2	8	12.5	0.178	2.22			0
				**Hemorrhoids**	DRR2	1			1.5		0.27			
16	*Echis carinatus sochureki* Stemmler, 1969, Sind Valley saw snake viper, Pathar Sap	Fat, oil	T	Snake bite	EC1	2	3	7	28.6	0.156	4.44	Snake bite	[48, 59]	0.25
				**Joint pain**	EC2	2			28.6		4.45			
				**Sexual potency**	EC3	2			28.6		4.45			
**Fishes**														
17	*Ctenopharyngodon idella* (Valenciennes, 1844), Grass carp, Grass carp	Brain, oil	O, T	Eyesight	CI1	51	5	51	100	1.000	100	Treat cold, enhance memory, energy and sexual power, joint pain	[31, 48]	0.20
				Night blindness	CI2	6			11.8		11.8			
				Fever	CI3	44			86.3		86.3			
				Cold	CI4	51			100		100			
				Joint pain	CI5	3			5.9		5.9			
18	*Cyprinus carpio* Linnaeus, 1758, Common carp, Gulfam	Brain, oil	O, T	Eyesight	CC1	5	5	62	8.1	1.000	8.1	CNS, erysipelas, lumbago, enhance memory, energyand sexual power, reduce overweight, and treat cold	[48, 60]	0.20
				Night blindness	CC2	5			6.8		6.8			
				Fever	CC3	60			96.8		96.8			
				Cold	CC4	3			4.1		4.1			
				Joint pain	CC5	3			4.1		4.1			
19	*Hypophthalmichthys molitrix* (Valenciennes, 1844), Silver carp, Silver carp	Brain, oil	O, T	**Eyesight**	HM1	5	5	60	8.3	1.000	8.3			0
				**Night blindness**	HM2	5			8.3		8.3			
				**Fever**	HM3	60			100		100			
				**Cold**	HM4	60			100		100			
				**Joint pain**	HM5	3			5.0		5			
20	*Cirrhinus mrigala* (Hamilton, 1822), Mrigal carp, Mori	Brain, oil	O, T	Eyesight	CM1	5	5	50	10.0	1.000	10	Reduces weight, joint pain, enhance memory, sexual power, provide energy, against cold	[48, 59]	0.40
				Night blindness	CM2	5			10.0		10			
				Fever	CM3	50			100		100			
				Cold	CM4	44			88.0		88			
				Joint pain	CM5	3			6.0		6			
21	*Cirrhinus reba* (Hamilton, 1822), Reba carp, Reba Machhali	Brain, oil	O, T	**Eyesight**	CR1	5	5	67	7.5	1.000	7.5			0
				**Night blindness**	CR2	5			7.5		7.5			
				**Fever**	CR3	67			100		100			
				**Cold**	CR4	55			82.1		82.1			
				**Joint pain**	CR5	3			4.5		4.5			
22	*Labeo rohita* (Hamilton, 1822), Rohu, Raho	Brain	O	Joint pain	LR1	55	8	90	61.1	1.000	61.1	Urine problem, stomachache, weakness, rheumatic pain, enhance memory, energy and sexual power, treat cold	[47, 48, 59, 61]	0.25
				Body pain	LR2	3			3.3		3.3			
				Sexual potency	LR3	7			7.8		7.8			
				Eye sight	LR4	5			5.6		5.6			
				Depression	LR5	2			2.2		2.2			
				Diabetes	LR6	2			2.2		2.2			
				Alzheimer	LR7	1			1.1		1.1			
				Heart disease	LR8	2			2.2		2.2			
23	*Labeo calbasu* (Hamilton, 1822), Orangefin labeo, Kalbans	Brain, oil	O, T	Joint pain	LC1	55	8	55	100	1.000	100	Increase energy, CNS , galactagogue, enhance memory, enhance energy, sexual power, reduce overweight, increase lactation in mother, energy, cold	[48, 62]	0.125
				Body pain	LC2	55			100		100			
				Sexual potency	LC3	55			100		100			
				Eye sight	LC4	5			9.1		9.1			
				Depression	LC5	2			3.6		3.6			
				Diabetes	LC6	2			3.6		3.6			
				Alzheimer	LC7	1			1.8		1.8			
				Heart disease	LC8	2			3.6		3.6			
24	*Labeo dero* (Hamilton, 1822), Dero, Dero machhali	Brain, oil	O, T	**Eyesight**	LD1	5	5	57	8.8	1.000	8.8			0
				**Night blindness**	LD2	5			8.8		8.8			
				**Fever**	LD3	57			100		100			
				**Cold**	LD4	57			100		100			
				**Joint pain**	LD5	3			5.3		5.3			
25	*Gibelion catla* (Hamilton, 1822), Catla, Thaila	Brain	O	Improve CNS	GC1	10	2	56	17.9	1.000	17.9	Increases energy & memory, galactagogue, rheumatic pain, enhance sexual power	[48, 59, 62]	1
				**Cold**	GC2	3			5.4		5.4			
26	*Channa punctata* (Bloch, 1793), Spotted snakehead, Dola	Flesh	O	Regulate blood chemical	CP1	4	4	58	6.9	1.000	6.9	Appetite, blood purification, malaria, body pain, enhance energy, sexual power, treat cold and joint pain	[48, 61, 63, 64]	0
				Eye sight	CP2	4			6.9		6.9			
27	*Channa marulius* (Hamilton, 1822), Great snakehead, Soul	Flesh	O	Sexual potency	CMH1	12			20.7	1.000	20.7	Increases sex power, hemoglobin, memory and energy, cure rheumatic pain, cold, joint pain	[30, 59, 61, 62]	0.5
				Weakness	CMH2	10			17.2		17.2			
28	*Oreochromis niloticus* (Linnaeus, 1758), Nile tilapia, Tilapia	Flesh ash	T	Scorpion, wasp, spider and insect bite	ON1	4	2	53	7.5	1.000	7.5	Abscesses, carbuncle, vision, scorpion bite, enhance memory, energy and sexual power	[48, 60]	0.5
				Skin Burn	ON2	12			22.6		22.6			
29	*Rita rita* (Hamilton, 1822), Catfish, Khaga	Flesh and oil	O, T	Joint pain	RR1	3	2	47	6.4	1.000	6.4	Joint pain, CNS, enhance energy, sexual power, treat cold and joint pain	[48, 59]	1
				Sexual potency	RR2	3			6.4		6.4			
30	*Bagarius bagarius* (Hamilton, 1822), Goonch, Foji Khaga	Flesh soup	O	Body pain	BB1	3	2	66	4.5	1.000	4.5	Body burns, body pain, stomach pain	[57, 61]	0.5
				Sexual potency	BB2	3			4.5		4.5			
31	*Mystus cavasius* (Hamilton, 1822), Gangetic mystus, Tangra Machhali	Flesh	O	Small pox	MC1	1	2	10	10.0	0.222	2.22	Small pox, joint pain	[59, 65]	0.5
				Chicken pox	MC2	1			10.0		2.22			
32	*Mastacembelus armatus* Skyes, 1839, Zig-zag eel, Baam Machhali	Flesh	O	**Sexual potency**	MA1	10	2	65	15.4	1.000	15.4			0
				**Weakness**	MA2	40			61.5		61.5			
33	*Wallago attu* (Bloch & Schneider, 1801),Wallago catfish, Mali	Flesh	O	Liver disease	WA1	2	2	68	2.9	1.000	2.9	Joint pain, dysentery, liver tonic, pile, enhance memory, sexual power, cure liver diseases, cold, joint pain	[48, 66–68]	0.5
				Hepatitis	WA2	3			4.4		4.4			
34	*Notopterus notopterus* (Pallas, 1769), Bronze featherback, But Pari	Flesh	O	Small pox	NN1	1	2	25	4.0	0.556	2.224	Chicken pox, pain	[69, 70]	0.5
				Chicken pox	NN2	1			4.0		2.224			
35	*Puntius sophore* (Hamilton, 1822), Spotfin swamp barb, Sophore popra	Flesh	O	Regulate blood-chemical balance	PS1	2	1	7	28.6	0.156	4.4616	Blood purification, cold	[63, 70]	1
36	*Pethia ticto* (Hamilton, 1822), Ticto barb, Ticto popra	Brain	O	**Night blindness**	PT1	3	4	5	60.0	0.111	6.66			0
				**Eye-sight**	PT2	5			100		11.1			
				**Improve CNS**	PT3	3			60.0		6.66			
37	*Heteropneustes fossilis* (Bloch, 1794), Stinging catfish, Sangehi machhali	Flesh	O	Increase hemoglobin level	HF1	2			40.0	0.111	4.44	Joint pain, increase hemoglobin, treat fever, pain, wound, enhance memory, energy, sexual power, reduces overweight	[47, 48, 52, 71, 72]	1
38	*Gagata cenia* (Hamilton, 1822), Indian gagata, Gagata cenia	Bone	O	**Urine problem**	GCH1	2	1	8	25.0	0.178	4.45			0

MA. Modeof application, IMA. Informant of major ailment, ND. Number of diseases, FC. Frequency of citation, FL. fidelity level, RPL. Relative popularity level, ROP. Rank order priority, SI. Similarity index, T. topical, O. oral

*Italic: Medicinal uses which are reported for very first time in this study

*Bold: Medicinal uses which are different from than reported uses

Likewise, 14 species of lizards had the suffix “kirli” such as *Laudakia melanura melanura* (kali kirli), *Eublepharis macularius* (korh kirli), *Cyrtopodion montiumsalsorum* (sahrai kirli), *Cyrtopodion Scabrum* (toor Kirli), *Hemidactylus flaviviridis* (gharailo kirli), *Hemidactylus persicus* (Irani kirli), *Acanthodactylus cantoris* (neeli poosh kirli), *Ophisops jerdonii* (safaid dahari kirli), *Ablepharus grayanus* (bahri kirli), *Ablepharus pannonicus* (surakh posh kirli), *Eutropis macularia* (bori kaa kirli), *Eurylepis taeniolatus taeniolatus* (maidani kirli), *Ophiomorus tridactylus* (tray ungl kirli), and *Scincella himalayana* (pahari kirli). Fifteen species of snakes had the suffix “sap,” e.g., *Leptotyphlops macrorhynchus* (dhaga sap), *Ramphotyphlops braminus* (dhaga sap), *Python molurus* (azdha sap), *Amphiesma stolatum* (lakeer dhari sap), *Boiga trigonata* (billi sap), *Lytorhynchus paradoxus* (ollu sap), *Oligodon arnensis arnensis* (kukri sap), *Oligodon taeniolatus taeniolatus* (kukri sap), *Platyceps rhodorachis rhodorachis* (Pheesi sap), *Psammophis leithii leithii* (teer maar sap), *Psammophis schokari schokari* (saharai sap), *Ptyas mucosus mucosus* (chohay-maar sap), *Xenochrophis piscator piscator* (chitra sap), *Daboia russelii russelii* (dabian wala sap), and *Echis carinatus sochureki* (pathar sap). Only two local names included “kukri sap” in the vernacular name (*Oligodon arnensis arnensis* and *O*. *taeniolatus taeniolatus*), and “dhaga sap” was the local name of *Leptotyphlops macrorhynchus* and *Ramphotyphlops braminus.* Local nomenclature of snakes was also based on their external morphology such as *L*. *melanura melanura*, *A*. *cantoris*, *A*. *pannonicus*, *Naja naja naja*, and *E*. *macularia* which have black, blue, red, and black and brown color lines and hence were named kali kirli, neeli poosh kirli, surakh posh kirli, Kala naag, and bori kaa kirli, respectively.

The vernacular names of the reported species had also connections with the habitats like sahrai kirli as the name of *C*. *montiumsalsorum*, because it lives in desert (Sahari) landscapes, whereas *H*. *flaviviridis* and *B*. *stomaticus* were named asghariallo daddo and ghariallo kirli, respectively as they live in houses (ghar) or their vicinity. *S*. *himalayana*is was called pahari kirli because it inhabits mountain areas (pahar). Likewise, vernacular names had also a connection with the morphology of species, *O*. *tridactylus*, e.g., has three tows and is known as “tray ungl kirli,” *L*. *macrorhynchus* and *R. braminus* snakes are very thin and are known as dhaga sap.

Eight species of fish had the same suffix “machhali” such as *Cirrhinus reba* (reba machhali), *Labeo dero* (dero machhali), *Oreochromis niloticus* (tilapia/chira machhli), *Mystus cavasius* (tangra machhali), *Mastacembelus armatus* (baam machhali), *Osteobrama cotio* (pali roo machhali), *Salmostoma bacaila* (choti chal machhali), and *Heteropneustes fossilis* (sangehi machhali). The English and local names of *Hypophthalmichthys molitrix* were the same—“silver carp”. Vernacular names of two species were based on their color: *H*. *molitrix* has silver color and *C*. *idella* has grass color; therefore, they were named as silver carp and grass carp, respectively. Some fish were also classified on the basis of morphology, e.g., the shape of *Channa punctata* is similar to an arm muscle; therefore, it was called dola (bicep muscle); *O*. *niloticus* size resembles a house sparrow andthus, the species was named chira machhli (chira is house sparrow) and *S*. *bacaila* has small size, and was known as choti chal machhali (choti means small).

### Myths about herptiles and fish

Some common myths on snake and fish species were also noted during the field survey. These myths were comparable to previous reports [15].

- It is a common assumption that if *Eryx johnii* (common sand boa) bites somebody, it will bite on the arrival of the new moon in each month.
- If the male or female ofa snake pair is killed by someone, then the other will certainly take revenge from the assassin.
- Naja naja changes into a human after 100 year of age and can transfer poison to a person if it sniffs someone.
- Some people of the study area believe that *Python molurus* (rock python) has seven mouths.
- Most people believe that a special bone called Mankana is present in the snake head, and that this bone can absorb poison from snake-bitten people.
- Seeing a snake in a dream means that an enemy may attack a person.
- Every species of fish has a special kind head like a human and body like a fish, which locally known as Jal Pari.
- Turtles have a blade which can cut anything.

### Ethnomedicinal uses herptiles and fish

**Fig. 3:**
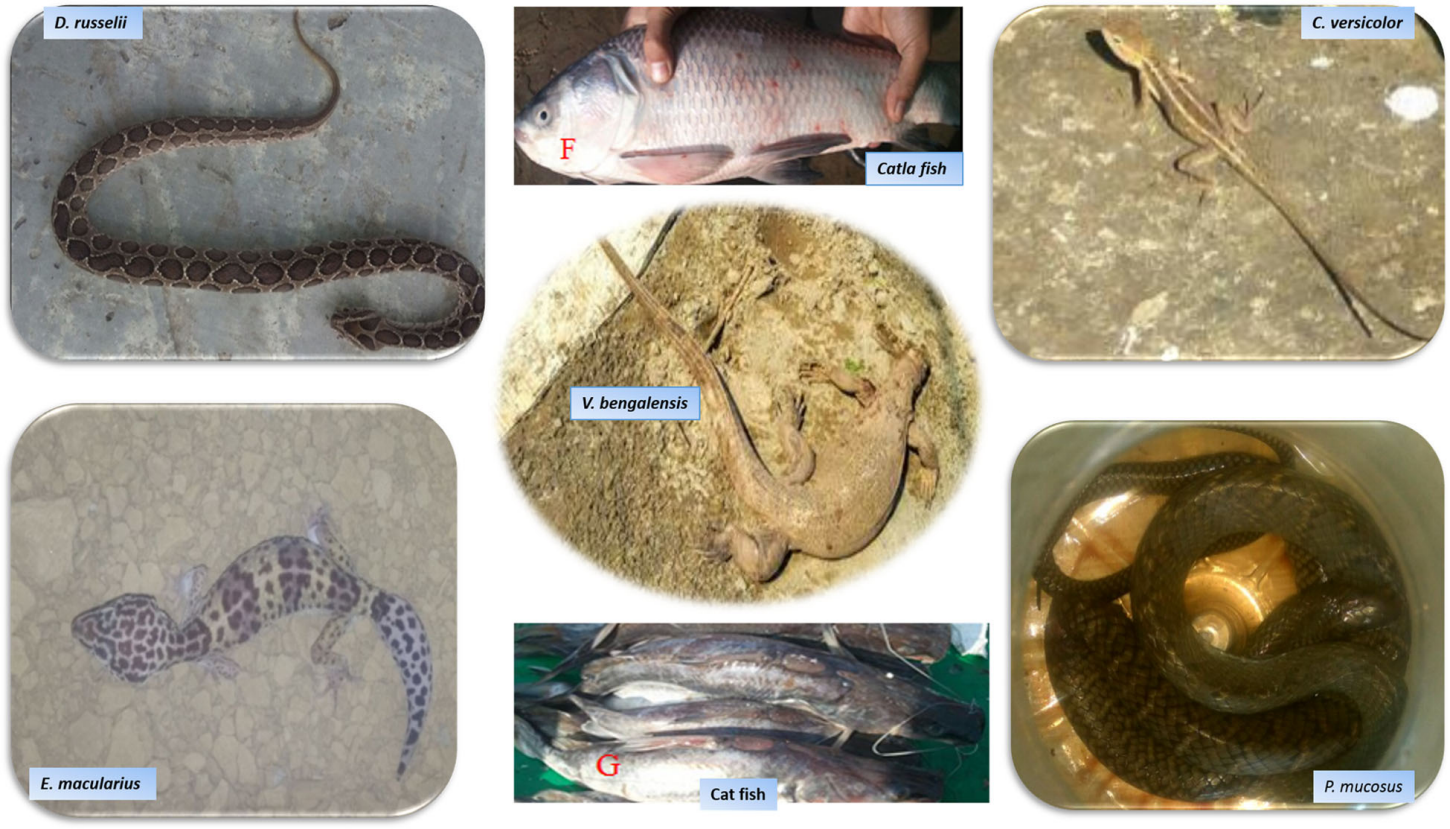
Herptiles and fish species used for medicinal purpose in the study area

Inhabitants of the study area possessed significant knowledge on the medicinal as well as cultural uses of animal species, particularly that of herptiles and fish (Fig. 3). As mentioned in Table 1, 38 species of herptiles and fish were used to treat various health disorders such as allergy, cardiovascular, nervous and respiratory disorders, sexual impotency, skin infections, and as antidote and anti-diabetic agents in human and livestock (Table 2).

**Table 2:** Cultural uses of herptiles and fishes in the study area

Sr. #	Scientific, common, and local name	FC	RFC	CU	STS	MD	NR	CC	TL	ET	FD	HF	MG	EX	OR	SPR
1	*Bufotes latastii (Boulenger, 1882), Ladakh Toad, Chitkbra daddo*	7	0.401	2	LC	X	√	X	X	X	X	√	X	X	X	X
2	*Duttaphrynus stomaticus* (Lütken, 1864),, Indus Valley toad, Ghariallo daddo	30	1.720	3	LC	√	√	X	X	X	X	√	X	X	X	X
3	*Microhyla ornata* (Dumerila and Bibron, 1841), Ant Frog, Bona daddo	7	0.401	2	LC	X	√	X	X	X	X	√	X	X	X	X
4	*Fejervarya limnocharis* (Gravenhorst, 1829), Asian Grass Frog, Pidda daddo	5	0.287	2	LC	X	√	X	X	X	X	√	X	X	X	X
5	*Hoplobatrachus tigerinus* (Daudin, 1802), Indian Bullfrog, Wada daddo	40	2.294	4	LC	√	√	√	X	X	X	√	X	X	X	X
6	*Sphaerotheca breviceps* (Schneider, 1799), Indian burrowing frog, Chota dahri daddo	5	0.287	4	LC	X	√	X	X	X	X	√	X	X	X	X
7	*Aspideretes gangeticus* Ernst and Barbour, 1989, Indian soft shell, Plaither	8	0.459	5	VU	√	√	√	X	X	X	√	X	X	X	√
8	*Aspideretes hurum* Ernst and Barbour, 1989, Peacock soft shell, Kachhokuma	8	0.459	5	VU	√	√	√	X	X	X	√	X	X	X	√
9	*Lissemys punctata andersoni* Webb, 1980, Indian Flap-shelled Turtle, Hara kachopra	7	0.401	5	LC	√	√	√	X	X	X	√	X	X	X	√
10	*Calotes minor* (Hardwicke and gray, 1827), Hardwicke's Short Tail Agama, Choti dum kirli	5	0.287	2	NE	X	X	X	X	X	X	√	X	X	X	√
11	*Calotes versicolor* (Daudin, 1802), Oriental garden lizard, Girgit	12	0.688	3	NE	√	X	X	X	X	X	√	X	X	X	√
12	*Laudakia agrorensis* (Stoliczka, 1872), Agror agama, Goh	20	1.147	4	NE	√	X	X	√	X	X	√	X	X	X	√
13	*Laudakia melanura melanura* (Blyth, 1854), Black agama, Kali kirli	2	0.115	2	NE	X	X	X	X	X	X	√	X	X	X	√
14	*Trapelus agilis pakistanensis* Rastegar-Pouyani, 1999, Brilliant ground agama, Korh kirla	5	0.287	2	NE	X	X	X	X	X	X	√	X	X	X	√
15	*Eublepharis macularius* (Blyth, 1854), Common leopard gecko, Korh kirli	6	0.344	4	NE	√	X	X	X	X	X	√	X	X	X	√
16	*Cyrtopodion montiumsalsorum* (Annandale, 1913), Salt range ground gecko, Sahrai kirli	4	0.229	2	NE	X	X	X	X	X	X	√	X	X	X	√
17	*Cyrtopodion Scabrum* (Heydenn 1827), Common tuberculated ground gecko, Toor kirli	2	0.115	2	LC	X	X	X	X	X	X	√	X	X	X	√
18	*Hemidactylus flaviviridis* Rüppell, 1835, Yellow belly common house gecko, Gharailo kirli	44	2.523	2	NE	X	X	X	X	X	X	√	X	X	X	√
19	*Hemidactylus persicus* Anderson, 1872, Persian house gecko, Irani kirli	3	0.172	2	NE	X	X	X	X	X	X	√	X	X	X	√
20	*Acanthodactylus cantoris* Gunther, 1864, Blue tailed sand lizard, Neeli poosh kirli		0.000	2	NE	X	X	X	X	X	X	√	X	X	X	√
21	*Ophisops jerdonii* Blyth, 1853, Punjab snake-eyed lacerta, Safaid dahari kirli	4	0.229	2	NE	X	X	X	X	X	X	√	X	X	X	√
22	*Ablepharus grayanus* (Stoliczka, 1872), Earless snake eyed skink, Bahri kirli	1	0.057	2	NE	X	X	X	X	X	X	√	X	X	X	√
23	*Ablepharus pannonicus* (Fitzinger, 1824), Red tail snake eyed skink, Surakh posh kirli	3	0.172	2	NE	X	X	X	X	X	X	√	X	X	X	√
24	*Eutropis macularia (Blyth, 1853), Bronz grass skink, Bori kaa kirli*	2	0.115	2	NE	X	X	X	X	X	X	√	X	X	X	√
25	*Eurylepis taeniolatus taeniolatus* Blyth, 1854, Alpine Punjab skink, Maidani kirli	3	0.172	2	NE	X	X	X	X	X	X	√	X	X	X	√
26	*Ophiomorus tridactylus*(Blyth, 1853), Three toed snake skink, Tray ungl kirli	2	0.115	2	NE	X	X	X	X	X	X	√	X	X	X	√
27	*Scincella himalayana* (Günther, 1864), Himalayan skink, Pahari kirli	2	0.115	2	NE	X	X	X	X	X	X	√	X	X	X	√
28	*Uromastyx hardwickii* Smith,1935, Indus spiny-tail lizard, Sanda	24	1.376	3	NE	√	X	X	X	X	X	√	X	X	X	√
29	*Varanus bengalensis* (Daudin, 1802), Bengal Monitor, Bengali goh, Goh	20	1.147	4	LC	√	X	X	√	X	X	√	X	X	X	√
30	*Leptotyphlops macrorhynchus* Hahn, 1978, long-nosed worm snake, Dhaga sap	5	0.287	2	LC	X	√	X	X	X	X	√	X	X	X	X
31	*Ramphotyphlops braminus* Daudin, 1803, Barhminy blind snake, Dhaga sap	3	0.172	2	NE	X	√	X	X	X	X	√	X	X	X	X
32	*Python molurus* (Linnaeus, 1758), Rock pathon, Azdha sap	5	0.287	4	VU	√	√	X	X	√	X	√	X	X	X	X
33	*Amphiesma stolatum* (Linnaeus, 1758), Buff Striped Keelback, Lakeer dhari sap	5	0.287	3	NE	X	√	X	X	√	X	√	X	X	X	X
34	*Boiga trigonata* (Schneider, 1802), Common cat snake, Billi sap	5	0.287	2	LC	X	√	X	X	X	X	√	X	X	X	X
35	*Lytorhynchus paradoxus* (Gunther, 1875), Sind longnose sand snake, Ollu sap	6	0.344	2	NE	X	√	X	X	X	X	√	X	X	X	X
36	*Oligodon arnensis arnensis* (Shaw, 1802), Banded kukri snake, Kukri Sap	6	0.344	2	NE	X	√	X	X	X	X	√	X	X	X	X
37	*Oligodon taeniolatus taeniolatus* (Jerdon, 1853), Streaked kukri snake, Kukri sap	4	0.229	2	LC	X	√	X	X	X	X	√	X	X	X	X
38	*Platyceps rhodorachis rhodorachis* (Jan, 1865), Cliff racer, Pheesi sap	5	0.287	2	NE	X	√	X	X	X	X	√	X	X	X	X
39	*Eryx johnii*(Russell, 1801), Common Sand boa, Do moi	22	1.261	3	NE	√	√	X	X	X	X	√	X	X	X	X
40	*Psammophis leithii leithii* Günther, 1869, Steppe ribbon snake, Teer maar sap	8	0.459	2	NE	X	√	X	X	X	X	√	X	X	X	X
41	*Psammophis schokari schokari* (Forskal, 1775), Schokari sand racer snake, Saharai sap	5	0.287	2	NE	X	√	X	X	X	X	√	X	X	X	X
42	*Ptyas mucosus mucosus* (Linnaeus, 1758), Indian rat snake, Chohay-maar sap	25	1.433	4	NE	√	√	X	X	√	X	√	X	X	X	X
43	*Xenochrophis piscator piscator* (Schneider, 1799), Chekered keelback, Chitra sap	8	0.459	2	NE	X	√	X	X	X	X	√	X	X	X	X
44	*Bungarus caeruleus caeruleus* (Schneider, 1801), Common krait, Sangchor sap	12	0.688	2	NE	X	√	X	X	X	X	√	X	X	X	X
45	*Naja naja naja* (Linnaeus, 1768), Indian cobra, Kala naag	17	0.975	5	LC	√	√	X	X	√	X	√	√	X	X	X
46	*Daboia russelii russelii* (Shaw and Nodder, 1797), Russell's chain viper, Dabian wala sap	8	0.459	3	NE	√	√	X	X	X	X	√	X	X	X	
47	*Echis carinatus sochureki* Stemmler, 1964, Sind Valley saw snake viper, Pathar Sap	7	0.401	3	NE	√	√	X	X	X	X	√	X	X	X	X
48	*Ctenopharyngodon idella* (Valenciennes, 1844), Grass carp, Grass carp	51	2.924	6	NE	√	√	√	√	√	√	X	X	X	X	X
49	*Cyprinus carpio (Linnaeus, 1758), Common carp, Gulfam*	62	3.555	6	VU	√	√	√	√	√	√	X	X	X	X	X
50	*Hypophthalmichthys molitrix* (Valenciennes, 1844), Silver carp, Silver carp	60	3.440	6	NT	√	√	√	√	√	√	X	X	X	X	X
51	*Cirrhinus mrigala* (Hamilton, 1822), Mrigal carp, Mori	50	2.867	6	LC	√	√	√	√	√	√	X	X	X	X	X
52	*Cirrhinus reba (Hamilton, 1822), Reba carp, Reba Machhali*	67	3.842	6	LC	√	√	√	√	√	√	X	X	X	X	X
53	*Labeo rohita* (Hamilton, 1822), Rohu, Raho	90	5.161	6	LC	√	√	√	√	√	√	X	X	X	X	X
54	*Labeo calbasu (Hamilton, 1822), Orangefin labeo, Kalbans*	55	3.154	6	LC	√	√	√	√	√	√	X	X	X	X	X
55	*Labeo dero (Hamilton, 1822), Dero, Dero machhali*	57	3.268	6	LC	√	√	√	√	√	√	X	X	X	X	X
56	*Gibelion catla (Hamilton, 1822), Catla, Thaila*	56	3.211	6	LC	√	√	√	√	√	√	X	X	X	X	X
57	*Channa punctata(Bloch, 1793), Spotted snakehead, Dola*	58	3.326	6	LC	√	√	√	√	√	√	X	X	X	X	X
58	*Channa marulius (Hamilton, 1822), Great snakehead, Soul*	58	3.326	6	LC	√	√	√	√	√	√	X	X	X	X	X
59	*Oreochromis niloticus* (Linnaeus, 1758), Nile tilapia, Tilapia/Chira machhli	53	3.039	6	NE	√	√	√	√	√	√	X	X	X	X	X
60	*Rita rita* (Hamilton, 1822), Rita, Khaga	47	2.695	6	LC	√	√	√	√	√	√	X	X	X	X	X
61	*Bagarius bagarius (Hamilton, 1822), Goonch, Foji Khaga*	66	3.784	6	NT	√	√	√	√	√	√	X	X	X	X	X
62	*Mystus cavasius* (Hamilton, 1822), Gangetic mystus, Tangra machhali	10	0.573	6	LC	√	√	√	√	√	√	X	X	X	X	X
63	*Mastacembelus armatus (Lacepède, 1800), Zig-zag eel, Baam machhali*	65	3.727	6	LC	√	√	√	√	√	√	X	X	X	X	X
64	*Sperata seenghala* (Sykes, 1839), Giant river-catfish, Sangari	62	3.555	5	NE	X	√	√	√	√	√	X	X	X	X	X
65	*Wallago attu* (Bloch & Schneider, 1801), wallago catfish, Mali	68	3.899	6	NT	√	√	√	√	√	√	X	X	X	X	X
66	*Eutropiichthys vacha (Hamilton, 1822), Batchwa vacha, Jhali*	56	3.211	5	LC	X	√	√	√	√	√	X	X	X	X	X
67	*Tor macrolepis* Heckel, 1838, Masheer, Masheer	40	2.294	5	NE	X	√	√	√	√	√	X	X	X	X	X
68	*Clupisoma garua (Hamilton, 1822), Garua bachcha, Bachhwa*	9	0.516	5	LC	X	√	√	√	√	√	X	X	X	X	X
69	*Notopterus notopterus (Pallas, 1769), Bronze featherback, But Pari*	25	1.433	6	LC	√	√	√	√	√	√	X	X	X	√	X
70	*Barilius modestusDay, 1872, Indus baril, Lahori Chalwa*	8	0.459	5	NE	X	√	√	√	√	√	X	X	X	X	X
71	*Puntius sophore (Hamilton, 1822), Spotfin swamp barb, Sophore popra*	7	0.401	6	LC	√	√	√	√	√	√	X	X	X	X	X
72	*Pethia ticto* (Hamilton, 1822), Ticto barb, Ticto popra	5	0.287	6	LC	√	√	√	√	√	√	X	X	X	X	X
73	*Parambassis ranga (Hamilton, 1822), Indian glassy fish, Ranga sheesha*	8	0.459	5	LC	X	√	√	√	√	√	X	X	X	√	X
74	*Sisor rabdophorus* Hamilton, 1822, Sisor catfish, Kirla machhali	7	0.401	5	LC	X	√	√	√	√	√	X	X	X	X	X
75	*Xenentodon cancila (Hamilton, 1822), Freshwater needlefish, Kaan Machhali*	5	0.287	5	LC	X	√	√	√	√	√	X	X	X	X	X
76	*Garra gotyla* (Gray, 1830), Gotyla, Pather Chat	7	0.401	5	LC	X	√	√	√	√	√	X	X	X	X	X
77	*Osteobrama cotio* (Hamilton, 1822), Cotio, Pali roo machhali	5	0.287	5	LC	X	√	√	√	√	√	X	X	X	X	X
78	*Salmostoma bacaila* (Hamilton, 1822), Large razorbelly minnow, Choti chal machhali	6	0.344	5	LC	X	√	√	√	√	√	X	X	X	X	X
79	*Heteropneustes fossilis* (Bloch, 1794), Stinging catfish, Sangehi machhali	5	0.287	6	LC	√	√	√	√	√	√	X	X	X	X	X
80	*Gagata cenia (Hamilton, 1822), Indian gagata, Gagata cenia*	8	0.459	6	LC	√	√	√	√	√	√	X	X	X	X	X
81	*Macrognathus pancalus* Hamilton, 1822, Barred spiny eel, Garoj	7	0.401	5	LC	X	√	√	√	√	√	X	X	X	X	X

*MD* medicinal, *NR* narrative, *CC* commercial, *TL* tools, *ET* entertainment, *FD* food, *HF* harmful, *OR* ornamental, *SPR* superstitious, *STS* status

### Body part(s) used

**Fig. 4:**
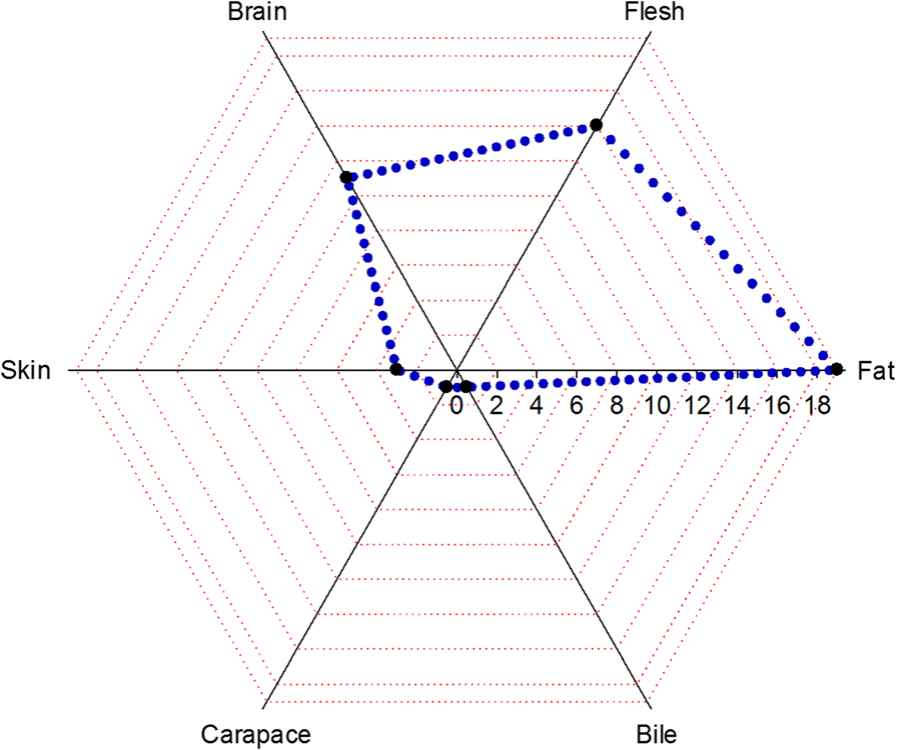
Body parts of herptiles and fish used in various recipes

In both herptiles and fish, as shown in Fig. 4, fat was the most commonly utilized body part and was used in the preparation of 19 recipes to treat a number of diseases, followed by flesh, brain, and skin, used in 14, 11, and 3 recipes, respectively. In the present study, the application of body fat of herptiles and fish served to treat rheumatic problems, eye diseases, sexual impotency, cold, fever, skin infections, nervous and cardiovascular diseases, diabetes, respiratory tract infections, urine problem, liver infections, heal wounds, and as antidote. This might be related to the presence of health beneficial metabolites, i.e., omega-3 fatty and omega-6 fatty acids, etc. It has been reported that these acids contribute substantially in the treatment of neurological disorders, thrombotic, atherosclerosis, and act as anti-aging agents [73]. Beneficial effects of omega-3 fatty acids on atherosclerosis are mainly through their actions on plasma lipids. Likewise, their role in the reduction of blood pressure and plasminogen activator inhibitor, improvement of metabolic syndrome, and maintain endothelial function may be due to other potential anti-atherogenic factors. As atherosclerosis is inflammatory diseases, omega-3 fatty acids offer protection through their anti-inflammatory effects [74–76]. The major benefit of omega-3 fatty acids in patients with type 2 diabetes mellitus is the enhancement in their highly atherogenic lipid profile. It has been reported less development of pancreatic islet-cell autoimmunity and reduction in insulin resistance was observed in mice treated with omega-3 fatty acids [75, 77].

Water-soluble vitamins [78], α1 and α2 collagen (I) proteins [79], and different amino acids, i.e., 4-hydroxyproline, aspartic acid, threonine, serine, glutamic acid, proline, glycine, alanine, cysteine, valine, methionine, methionine, leucine, tyrosine, phenylalanine, histidine, lysine, arginine, and hydroxylysine [80], have also been reported in the skin of fish. Collagen (I) is used in membranes for guided tissue regeneration [81]. While, essential amino acids profile of fish is required to humans for balanced diet [82].

Inhabitants of the study area use carapace of the turtle to treat internal injuries, allergy, cough, and as a sexual stimulant. These pharmacological properties of turtle carapace are mainly due to the presence of β-pleated sheet keratin [83], keratinocytes, melanocytes, lipids [84], and mineralized collagen fibrils [85]. Likewise, fish brain is used to treat joint pain, Alzheimer, heart diseases, for sexual potency, to improve eye-sight, as an anti-depressant and anti-diabetic. The health beneficial properties of fish brain mainly attributed to rich compounds of docosahexaenoic acid, omega-3 fatty acids, and proteins present in it [76, 86].

### Diseases treated

**Fig. 5:**
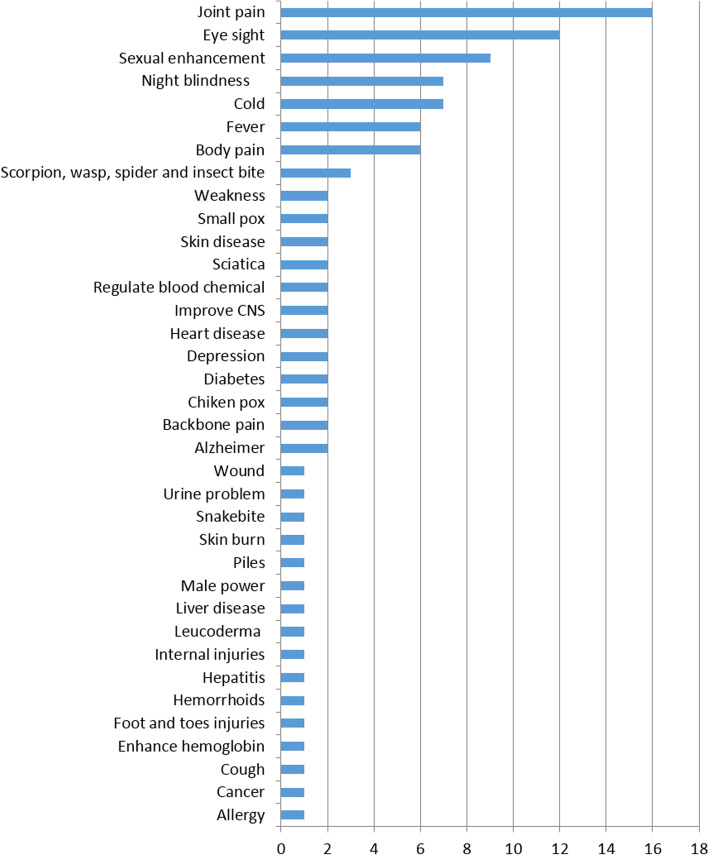
Number of recipes and diseases treated

As mentioned in Fig. 5, joint pain, eye diseases, sexual impotency, common cold, and fever were among the top ranked diseases treated with maximum number of animal-based recipes. Lack of hygiene, nutritional deficiency, and “community evils” were among the major factors involved in the high prevalence of diseases in the study area. Comparative analysis of the present findings with previous reports on medicinal uses of herptiles and fish species indicate that different methods of treatments and body parts were used in study area (Table 1). The inhabitants of the study area use skin of Indus valley toad (*Duttaphrynus stomaticus*) to treat skin infections, while the same species have been reported to treat allergy, thelitis, bolianerengia, bronchial pneumonia, dermatitis, abscess, and to heal wounds [46–48]. Body fat of the Indian bullfrog (*Hoplobatrachus tigerinus*) was used to treat backbone pain, sexual impotency, and joint pain; but in previous studies [49–52], different body parts of this species have been reported against acidity, burn, cold, cough, diarrhea, dysentery, and to heal wounds. In the study area, carapace ash, fat, and oil of the Indian flap-shelled turtle (*Lissemys punctata andersoni*) were used to enhance sexual potency and in the treatment of internal injuries, allergy, and cough. However, this species has also been reported to treat acne, piles, arthritis, asthma, bronchitis, burns, cough, dermatitis, epilepsy, backbone pain, diabetes, urinary obstruction, diarrhea, indigestion, lung diseases, malaria fever, menorrhagia, rashes, wound healing, and tuberculosis [48, 53–55]. Fat, oil, and bile of Agror agama (*Laudakia agrorensis*) were used for joint pain; sexual potency; snake, spider, wasp, and scorpion sting; as well as body pain. Same species are used to treat jaundice, joint pain, malaria, arthritis, burn, cough, fever, and skin disease [47, 48, 50, 52, 56–58]. The Indus spiny-tail lizard (*Uromastyx hardwickii*) is used in the treatment of body pain, joint pain, sciatica pain, and for sexual potency, whereas [48, 59] it was reported that the same species is useful against ear pain, backache, joint pain, and headache. Local people used body fat, skin, and oil of the Indian cobra (*Naja naja naja*) to treat sciatica, snakebite, to improve eye sight, and as sex stimulant. This species has been reported to cure arthritis, cancer, leprosy, muscular pain, as aphrodisiac, and as antidote [48, 51, 52, 59]. Fat and oil of Russell’s chain viper (*Daboia russelii russelii*) were used as a remedy for urine problem and hemorrhoids. However, in previous studies [50, 59, 87], different parts of this species have been reported as used against weak eye sight, urination, stool, flatus, and as anti-venom. Likewise, body fat and oil of the Sind valley saw snake viper (*Echis carinatus sochureki*) were used to treat joint pain, snakebite, weak eye sight, and to enhance sexual desire [48, 59].

Inhabitants of the study area preferred *Ctenopharyngodon idella* for the treatment of eyesight, night blindness, fever, cold, and joint pain, while the same species was reported to treat erectile disinfection, cold, to enhance memory, and sexual power and showed 0.20 similarity index with previous reports [31, 48]. Our findings revealed that *Cyprinus carpio* and *Cirrhinus mrigala* were effective against weak eyesight, night blindness, fever, cold, and joint pain. In previous studies [48, 60], *C*. *carpio* has been reported as used for CNS, erysipelas, lumbago, to enhance memory, enhance energy, sexual power, to reduce overweight, and against cold and has depicted similarity index = 0.20. Likewise, *C*. *mrigala* was reported to reduce weight, to treat joint pain, to enhance memory and sexual power, to provide energy, and to treat against cold [48, 59]. *Labeo rohita* and *L*. *calbasu* were used for the treatment of joint pain, body pain, sexual potency, eye-sight, depression, diabetes, Alzheimer, and heart diseases. Similarly, the fish species *Gibelion catla*, *Rita rita*, *Puntius sophore*, and *Heteropneustes fossilis* were used to enhance hemoglobin, regulate blood chemistry, joint pain, sexual potency, improve CNS, cold, and have highest similarity index 1 with previous reports [47, 48, 59, 61].

The ethnomedicinal uses of eight herptiles, i.e., *A*. *gangeticus*, *A*. *hurum*, *E*. *macularius*, *V*. *bengalensis*, *P*. *molurus*, *E*. *johnii*, *P*. *mucosus mucosus*, *D*. *russelii russelii* and five fish species including *H*. *molitrix*, *C*. *reba*, *L*. *dero*, *M*. *armatu*, and *P. ticto* were reported for the first time from this region, and showed zero similarity with other studies. Among herptiles, *H*. *tigerinus*, *D*. *stomaticus*, and *P*. *mucosus mucosu* and in fishes *L*. *rohita*, *W*. *attu*, and *C*. *reba* were top ranked with maximum informant reports, frequency of citations, and rank order priority.

### Cultural values of herptiles and fish

Cultural values of the reported species of herptiles and fish are given in Table 2. Local people of the study area used the skin of the black cobra in magic. Likewise, different species of snakes like the Indian cobra (*Naja naja naja*), Indian rat snake (*Ptyas mucosus mucosus*), and buff striped keelback snake (*Amphiesma stolatum*) were used for pleasure of the public such as the mongoose competition with a snake. According to local informants, the presence of the yellow belly common house gecko (*Hemidactylus flaviviridis*) in a home is considered as bad omen for residents. The Bengal monitor (*Varanus bengalensis*) is tight knot with rope and with help of that rope a person can climb walls. Fish species were not only used in the treatment of various diseases but also as nutritious food. Local inhabitants used fish flesh as bait for varieties of fish species from rivers, as reported earlier by [88].

Inhabitants of the study area used different species of fish for commercial purposes. Likewise, Indian soft shell, Peacock soft shell, Indian Flap-shelled Turtle, and Indian Bullfrog were captured and sold for lab practice. Only four species of herptiles such as Indian soft shell (*Aspideretes gangeticus*), Peacock soft shell (*Aspideretes hurum*), Indian Flap-shelled Turtle (*Lissemys punctata andersoni*), and common leopard gecko (*Eublepharis macularius*) were exported from the area, and are used as food and for medicines. Two species of fish, i.e., Bronze featherback (*Notopterus Notopterus*) and Indian glassy fish (*Parambassis ranga*), are ornamental fish for aquaria.

**Fig. 6:**
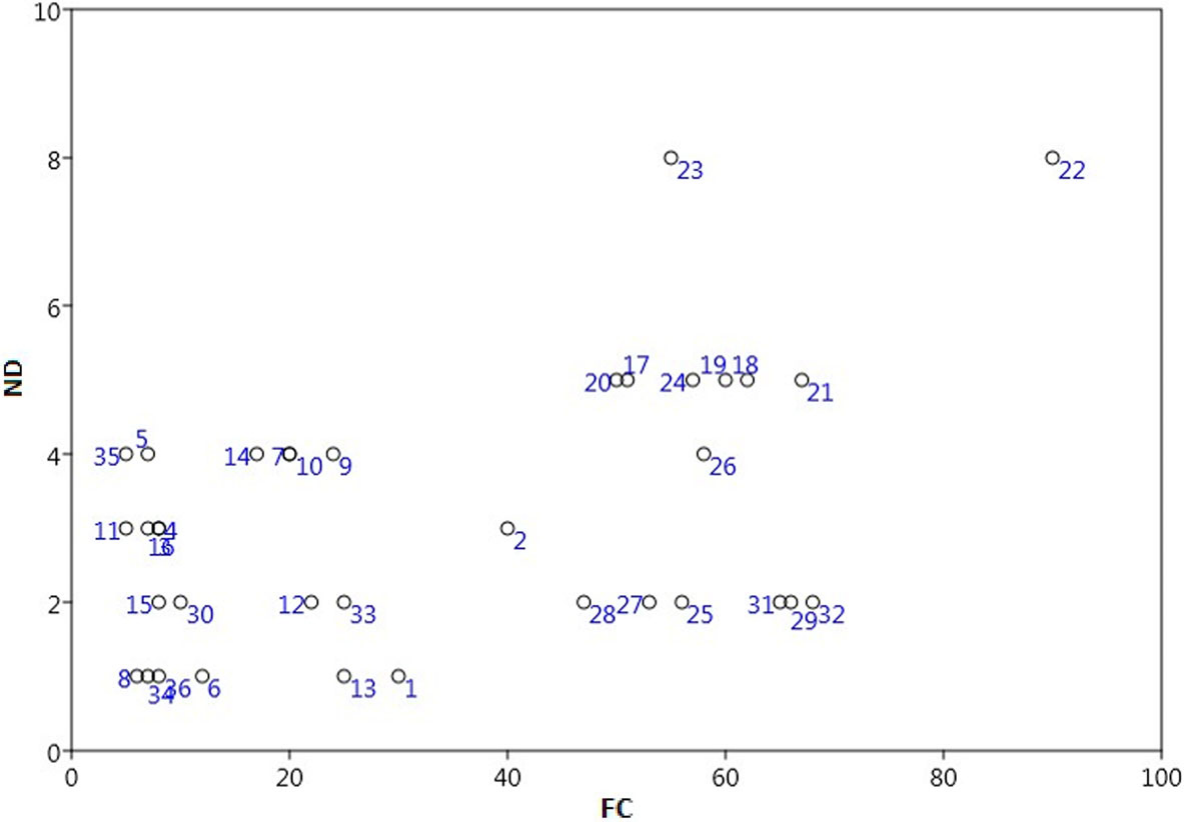
The relationship between numbers of diseases (ND) and frequency of citation (FC) in the study area

The animal species reported by the maximum number of respondents were frequently used to treat various diseases, and exhibited high FC (Frequency of Citation) ranging from 5 to 90 (Fig. 6), i.e., *Labeo rohita* (rohu) had a maximum FC (90), followed by *Wallago attu* (wallago catfish) and *Cirrhinus reba* (Reba carp) (68 and 67, respectively).

The fidelity level is utilized to recognize species that are commonly favored by people to treat different diseases [89, 90]. The FL of herptile and fish species in this study varied from 2.9 to 100% (Table 2). Nine species, including *U*. *hardwickii* (Indus spiny-tail lizard), *C*. *idella* (grass carp), *H*. *molitrix* (silver carp), *L*. *dero* (*dero*), *C*. *mrigala* (mrigal carp), *C. reba* (reba carp), *L*. *rohita* (rohu), *L*. *calbasu* (orange fin labeo), and *P*. *ticto* (ticto barb) which were used for sexual potency, fever, cold, and to treat eyesight, body pain, and joint disorders, depicted 100% FL (Fig. 5). These findings indicate the prevalence of particular diseases in the area that were cured with species having high FL. The animal species with maximum FL were highly used in the area, as compared to species having low FL. The FL of herptile and fish species was documented for the first time, and species with highest FL might be subjected to in-depth compositional analysis and bioactivities in pharmaceutical industries, as possible sources to manufacture novel drugs.

**Fig. 7:**
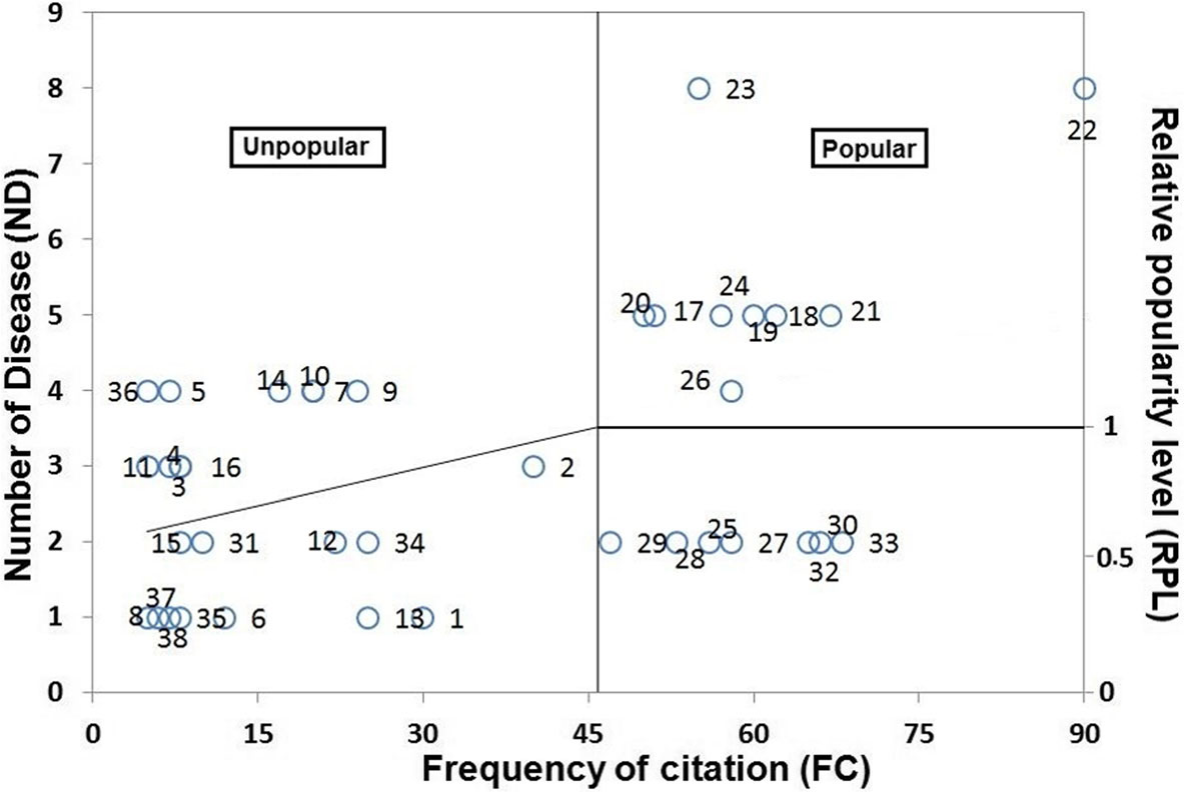
The relationship between informant numbers and the percentage of informants who argued similar use for that herpetofauna and fish; circled numbers show herpetofauna and fish names as given in Table 1

The relative popularity level (RPL) of the reported species is given in Table 2. Both herptile and fish species were classified as popular and unpopular categories based on RPL (Fig. 7), which were comparable to [43, 44]. During the study, we noted that *C*. *idella*, *C*. *carpio*, *H*. *molitrix*, *C*. *reba*, *C*. *mrigala*, *L*. *rohita*, *L*. *calbasu*, *L*. *dero*, *G*. *catla*, *C*. *punctata*, *C*. *marulius*, *O*. *niloticus*, *R*. *rita*, *B*. *bagarius*, *M*. *armatus*, and *W*. *attu* were most popular with RPL = 1.0 while all other species were ranked as unpopular. The high popularity of these species might be attributed to their high efficiency utilized as medicine.

The healing potential of herptile and fish species was observed using FL values, while rank order priority (ROP) was utilized to assign a suitable grade to all species with various FL values. The measured levels of ROP of each herptile and fish species are cited in Table 3. The ROP of seven species used to treat different diseases was above 100. These species were *C*. *idella* (eyesight and cold); *C*. *carpio* (fever); *H*. *molitrix* and *C. reba* (fever and cold); and *L. rohita*, *L*. *calbasu*, and *L. dero* used to treat joint pain, fever, and cold and for sexual potency. A decrease in ROP may be due to the decline in the popularity of ethnomedicinal and ethnocultural uses of fauna among local peoples. Moreover, the respondents of the rural area have more information and interaction with cultural and medicinal uses of animals compared to urban areas. The findings of the present survey were analogue to previous results for medicinal species of animal species in Palestine [44].

This study, for the first time, reported the use of flesh ash, fat, and oil of *Aspideretes gangeticus* (for skin diseases and sexual potency), *Aspideretes hurum* (backbone/joint pain), flesh ash of *Calotes versicolor* (for foot and toe injuries), fat oil of *Daboia russelii russelii* (to treat urinary problems and hemorrhoids), brain oil of *Hypophthalmichthys molitrix* (to improve eyesight, night blindness, and to treat fever, cold, joint pain). In addition, the brain oil of *Cirrhinus reba* and *Labeo dero* was used to treat eyesight, night blindness, fever, cold, and joint pain; the flesh of *Mastacembelus armatus* was used to improve sexual potency and body weakness; the brain of *Pethia ticto* was used to treat night blindness, eyesight, and to develop central nervous system; and the brain of *Gagata cenia* was used to treat urinary problems (Table 1).

Zoonoses with a wildlife reservoir are a major public health issue, affecting the whole world. Various pathogens and different modes of transmission are present, and many variables impact the epidemiology of different zoonoses. The recognition and importance of wildlife as a reservoir of zoonoses are increasing [91, 92]. The prevalence of transmission of disease-producing driving forces from fish to humans is however very low. In general, humans contract fish-borne disease through ingestion of tissues, or by contamination of the skin [93]. Human sensitivity to amphibian proteins in a laboratory setting is rare. It remains possible, however, to become sensitized to amphibian proteins through inhalation or skin contact [94].

### Conservation status of the reported species

Knowing the background of interaction and exploitation between humans and natural resources is vital for the implementation and development of animal and landscape conservation strategies [95]. Ethnozoological studies provide necessary information and contribute significantly to animal conservation because in addition to incorporating biological factors, and providing traditional knowledge on medicinal and cultural values of animal species in any area, such studies also cover cultural, social, economic and traditional roles of fauna in human civilization [96].

Based on the cultural uses of herptiles and fish species (Table 2), it was observed that 47% of the reported species are listed as least concern (LC), 44% are not evaluated (NE), 0.04% species (i.e., Indian soft shell, Peacock soft shell, Rock pathon, and Common carp) are vulnerable (V), and 0.03% species (i.e., Silver carp, Foji Khaga, and wallago catfish) are listed as near threatened (NT) globally by the International Union of Conservation of Nature (IUCN). Interestingly, most of the herptiles and fish species (74/91%) showed threats, and only 9% of the species were listed as threatened by IUCN as mentioned above. Use of animal species in traditional therapies and for cultural purpose by humans is not the only threat to animal biodiversity in any region. Factors also include changes in climate and various types of interactions in an ecosystem, i.e., food chain, food webs also contribute significantly in threatening animal population and diversity [96, 97]. Given the great need to find solutions to deal with the current crisis of biodiversity loss [98], more specifically that of animal species, it is obligatory to adopt strategies that address the problem in all its complexity. And for this, ethnozoology presents itself as an interdisciplinary tool, approaching the issue in an additional comprehensive method [99].

### Principal component analysis

**Fig. 8:**
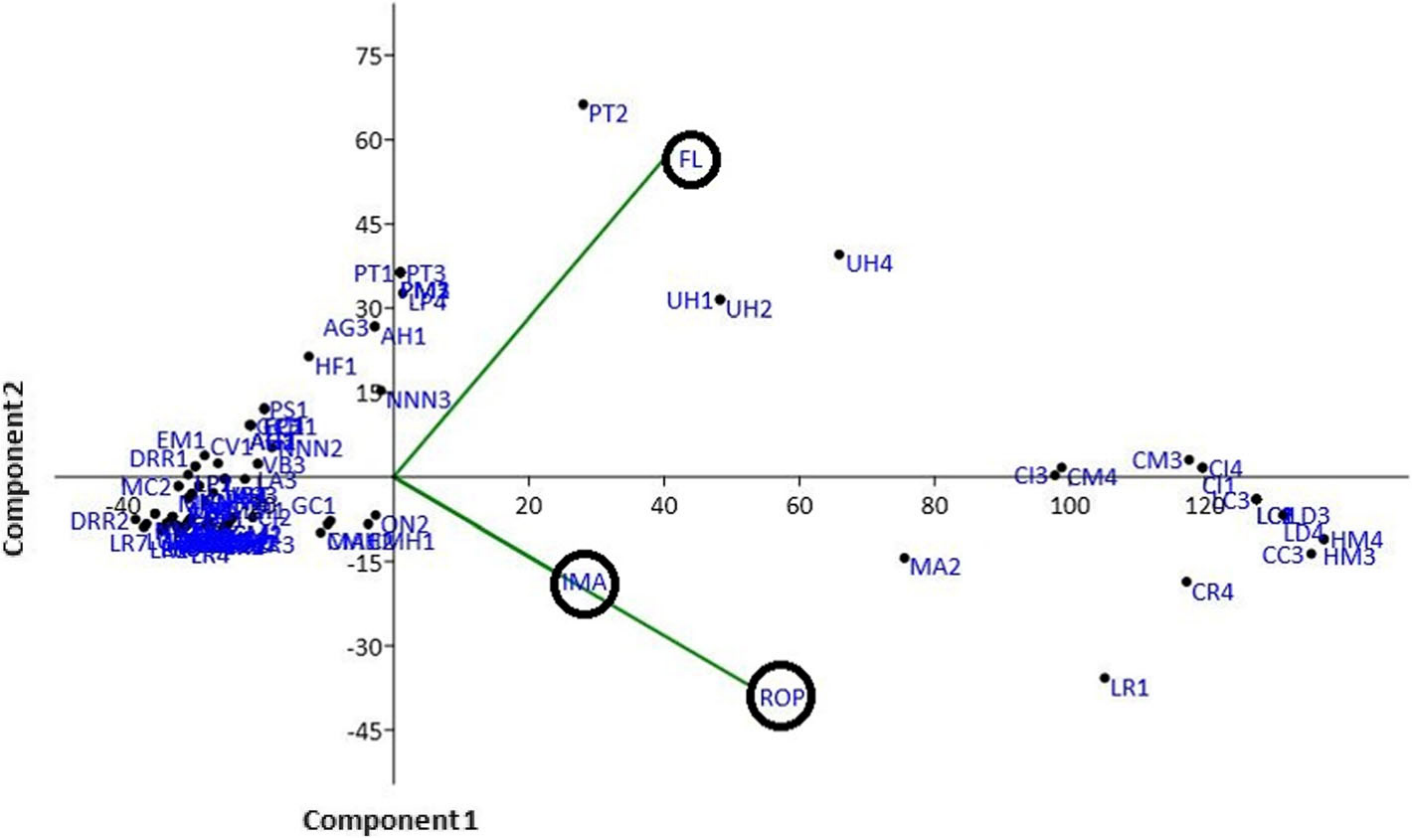
Principal components analysis (PCA) (code are present in Table 1). The positions of the arrows relative to components 1 and 2 show how strongly independent variables (IMA, ROP and RI) are correlated

The ethnomedicinal data were analyzed through principal component analysis (PCA), which allowed for the ordination of plots in terms of three variables viz. Informant of major ailment (IMA), fidelity level (FL), and rank order priority (ROP). The result of the PCA showed the sum of all the eigenvalues with total inertia of 3105.67. The first eigenvalue was high (2881.04) which showed high gradient strength in distribution of indigenous knowledge along the first axis (PC1). The total variation explained along this axis is (92.77%). The first two axes of the principal component analysis showed 99.99% variation in samples (component 1: 92.77%; component 2: 7.23%); therefore, only two axes were considered in Fig. 8. The variables IMA (*r* = 0.33506), FL (*r* = 0.57662), and ROP (*r* = 0.74514) positively correlated with first axis (PC 1) while IMA (*r* = − 0.23734) and ROP (*r* = − 0.52551) was negatively correlated with component 2 and FL (*r* = 0.81701) was positively correlated with component 2 (PC 2), which were comparable with previous studies [100].

## Conclusion

Traditional uses of various herptiles and fish species were recorded, and to the best of our knowledge, the ethno-pharmacological applications of 11 herptiles and seven fish species were reported for the first time from this region. Our findings revealed that the indigenous communities of the study possess significant traditional knowledge because of their strong relation with the nearby fauna. These results could be valuable for sustainable utilization and conservation of animal species. Additionally, detailed investigations on pharmacologically active substances and in vitro and/or in vivo of biological activities of compounds from herptiles and fish species with highest FL and FC could be interesting for the development of novel animal-based drugs to treat various health disorders.

## Data Availability

All data have already been included in the manuscript.
